# Accidental Death of Physicians

**Published:** 1853-05

**Authors:** 


ACCIDENTAL DEATH OF PHYSICIANS.

Among the victims of the Rail Road disaster, at Norwalk, were several physicians, members of the American Association returning from the meeting at New York. Some of them were men of note, and all were men of influence in their respective communities. Dr. Pierson, of Salem, Mass., was President of the Essex South District Medical Society. Dr. J. W. Smith, of Springfield, Mass., son of the late Dr. Nathan Smith, and brother of Prof. N. R. Smith, of Baltimore, held a high place in the public estimation. Dr Josiah Bartlett, of New Hampshire, was eminent in his profession. The others, Dr. Beach, and Dr. Welsh, of Connecticut, Dr. John G. Gray, and Dr. Dwight, of Mass., though less known, were men of moral worth as well as professional distinction, and their untimely death has carried sorrow and dismay into the neighborhoods where they had exerted their beneficent skill.

